# First multilocus sequence typing (MLST) of *Giardia duodenalis* isolates from humans in Romania

**DOI:** 10.1186/s13071-020-04248-2

**Published:** 2020-07-31

**Authors:** Carmen Costache, Zsuzsa Kalmár, Horațiu Alexandru Colosi, Alina Mihaela Baciu, Răzvan Vlad Opriş, Adriana Györke, Ioana Alina Colosi

**Affiliations:** 1 Department of Molecular Sciences, Discipline of Microbiology, Iuliu Hațieganu University of Medicine and Pharmacy, 6 Louis Pasteur Street, 400349 Cluj-Napoca, Romania; 2 Department of Parasitology and Parasitic Diseases, Faculty of Veterinary Medicine, University of Agricultural Sciences and Veterinary Medicine Cluj-Napoca, 3–5 Calea Mănăştur, 400372 Cluj-Napoca, Romania; 3 Department of Medical Education, Discipline of Medical Informatics and Biostatistics, Iuliu Hațieganu University of Medicine and Pharmacy, 6 Louis Pasteur Street, 400349 Cluj-Napoca, Romania

**Keywords:** *Giardia duodenalis*, Giardiasis, Multilocus genotyping, Assemblage, Subtypes, Romania, Parasitic diseases

## Abstract

**Background:**

*Giardia duodenalis* is one of the most prevalent and highly diverse human parasites, encompassing a complex of eight genetically distinct assemblages, each further divided into sub-assemblages. While in recent years, *G. duodenalis* genotype distribution patterns in humans have been intensely studied, there is still very little information available on the diversity of *Giardia* genotypes and sub-assemblages infecting people in Romania. In the present study, we investigated the genetic diversity of *Giardia duodenalis* in asymptomatic patients from Romania.

**Methods:**

Over an 11-month period, human feces from 7805 healthy adults were screened by microscopic analysis for *G. duodenalis* cysts during their obligatory periodic check-ups. DNA extraction was performed from microscopic-positive fecal samples, followed by multilocus sequence typing of four genetic loci of the ITS region, *gdh*, *tpi* and *bg* genes, followed by DNA sequencing and phylogenetic analysis. Statistical analysis was performed using EpiInfo 2000 software.

**Results:**

The prevalence of giardiasis in the present study was 0.42% (33/7805). Twenty-three samples (76.67%) were successfully genotyped at each locus. The *bg* and *tpi* genes had the highest typing success rate (100%). The identified assemblages were assemblage A in 27 cases (subtypes A2 and A3), and B in 3 cases.

**Conclusions:**

To our knowledge, the present study is the first report of multilocus sequence typing of *G. duodenalis* isolated from humans in Romania. The present results may shed light on *G. duodenalis* infection in humans at a regional and national level, thus increasing awareness against this parasitic infection. 
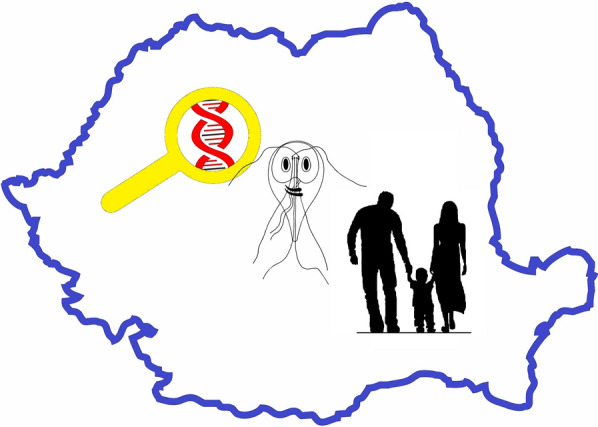

## Background

Foodborne diseases represent a serious public health concern that greatly impedes economic and social development in both developed and developing countries [1]. The non-invasive flagellated protozoan *Giardia duodenalis* (synonyms *Giardia intestinalis* and *Giardia lamblia*) has been ranked by the Food and Agriculture Organization (FAO) as the 11th most important food-borne pathogen [2]. *Giardia* has a global distribution, with human contamination occurring in both tropical and temperate areas. It remains the most frequently identified parasite from human fecal samples and the most common cause of parasitic gastroenteritis [3], registering annually around 280 million new cases worldwide [4]. Infection with *G. duodenalis* is the most frequently diagnosed gastrointestinal parasitic disease in Romania [5].

Human infection most often occurs by fecal-oral route through consumption of infested foods and water, by cysts (the resistant and infectious form of the parasite), and less frequently, through sexual practices (anal-oral sex). The cyst is largely resistant to environmental factors, thus contributing to its ability to infect animals and humans alike for months [6]. *Giardia duodenalis* infection can appear as endemic (mainly in subtropical and tropical regions), water-related epidemic and travel-related epidemic (accounting for 2–3% of traveler’s diarrhea) [7].

Infection with *G. duodenalis* can present as asymptomatic or symptomatic, acute or chronic. The clinical presentation of giardiasis is greatly influenced by the host’s immune response, duration of infection, virulence and the infective dose of the parasite, with the main symptoms including nausea, diarrhea (followed by dehydration), abdominal pain, vomiting and bloating [8, 9]. The evolution and severity of an infectious disease greatly depend on the interaction between the host factors and the virulence factors expressed by the etiological agent [10]. The expression of the virulent features of the parasite results from their genotype (e.g. assemblage). Currently, efforts are being made to correlate genetic traits to infectivity, routes of transmission and clinical symptoms [9, 11].

Despite its importance in the etiology of parasitic diarrheal disease, there is little, and sometimes contradictory information about the incidence of giardiasis in developing countries. The burden of the disease is usually estimated in these countries at the level of symptomatic groups, but the real prevalence and incidence in the general population remain largely unknown. The prevalence of *G. duodenalis* is influenced to a great extent by the diagnostic methods that are employed and by the expertise of medical professionals who participate in the diagnosis [12–14].

Besides humans, *Giardia* infects more than 40 other animal species [15]. Currently, eight morphologically distinct and valid species of *Giardia* have been described [8, 16, 17]. *Giardia duodenalis* is a genetically heterogenic parasite, and in relation to its hosts, this protozoan infects a wide range of mammalian species. Eight genetic groups, referred to as assemblages or genotypes (A to H) have been described [18], with assemblages A and B being the predominant human pathogens [9]. However, infections with assemblages C, D, E and F have also been identified in humans from Thailand (assemblages C and D), Egypt (assemblage E) and Ethiopia (assemblage F) [19]. Assemblage A has also been commonly reported in pets and livestock, while assemblage B is reported, as the dominant genotype in a smaller number of animal species. Due to their extended host specificity, both A and B assemblages are considered zoonotic pathogens [19, 20]. Studies on the worldwide prevalence of *Giardia* assemblages indicate that assemblage B is more often implicated in human infections (approximately 58%) than assemblage A (approximately 37%) [18]. However, it is important to note that the majority of these studies focused on symptomatic patients. This, coupled with the observation that assemblage A is more often found in asymptomatic patients [21, 22], indicates that the real prevalence of the two assemblages remains largely unknown. Additionally, depending on the region, the distribution of the two assemblages varies greatly. For example, while in Canada, Uganda and South Korea, studies have reported only assemblage A [23], a study in India has identified only assemblage B [24].

In Romania, the prevalence of human giardiasis varies from 2% to 27% in symptomatic patients, depending on the county [25]. Despite the high prevalence of this infection, the genetic characterization of the parasite has been documented only from animal fecal samples [26, 27] and water sources [28]. The present study aimed to investigate the molecular prevalence and genetic diversity of *G. duodenalis* from human isolates through multilocus sequence typing (MLST) of four genetic loci: β-giardin (*bg*); the glutamate dehydrogenase (*gdh*); the triosephosphate isomerase (*tpi)* genes; and the internal transcribed spacer (ITS) region of the ribosomal unit (ITS1-5.8S-ITS2). To our knowledge, this is the first molecular characterization of *G. duodenalis* identified in human stool samples from Romania.

## Methods

### Sample collection

All of the fecal samples included in the present study were collected by private laboratories (from Cluj-Napoca city, Cluj County, Romania) that were employed to carry out mandatory periodic check-ups. The laboratories in question serve both rural and urban areas in the western, north-western and central regions of Romania. Samples were then analyzed for the presence of *G. duodenalis* cysts at the Department of Microbiology, “Iuliu Hatieganu” University of Medicine and Pharmacy (Cluj-Napoca, Romania).

Between May 2018 and March 2019, 7805 healthy adults were screened for *G. duodenalis* during their mandatory periodic check-ups. Adults included in the study were apparently healthy, with no clinical suspicion of giardiasis. Stool samples (*n* = 3) from each subject were collected every 2 days (days 1, 3 and 5), in a sterile plastic container, void of preservatives. However, from the patients (*n* = 28) in which *G. duodenalis* was detected in the first stool sample, the second and the third sample was not collected, and from the patients (*n* = 5) in which *G. duodenalis* was detected in the second sample, the third sample was not collected. Each sample was kept at 4 °C and examined by light microscopy within 8 h of collection [29]. DNA extraction was carried out within 2 months of sample collection.

### Microscopic analysis

The fecal samples were analyzed for the presence of *G. duodenalis* cysts by direct microscopic examination of a wet mount. Prior to examination, each sample was concentrated by flotation technique and stained with 2% Lugol iodine solution [30, 31]. The wet mount was examined under a light microscope (Zeiss, Jena, Germany), using the 20× and 40× objectives to screen the entire sample area. Microscopic-positive samples were vortexed and stored in 95% ethanol (1 part sample, 4 parts ethanol) at − 20 °C [32].

### DNA extraction and PCR analysis

DNA extraction was performed using Isolate Fecal DNA kit (Bioline, London, UK) from *Giardia*-positive samples confirmed by microscopic examination. All isolates were investigated at three coding genes (*gdh*, *bg* and *tpi*) and the ITS region. The amplification was performed on a T100 Thermal Cycler (Bio-Rad, California, US) using the 2× Red PCR Master mix (Rovalab, Teltow, Germany) without addition of DMSO. In all cases, nested-PCR (nPCR) was performed in a final volume of 25 µl using 10 µM of each primer (GeneriBiotech, Hradec Králové, Czech Republic). In the first PCR reaction 1 µl of template DNA, while in the second reaction, 1 µl of template from the first-round PCR was used. Cycling conditions and primers are detailed in Table 1.

**Table 1:** Primers and PCR conditions

Gene	PCR reaction	Product length (bp)	Primer name	Primer (5’-3’)	PCR conditions	Reference
*bg*	1st	753	G7	AAGCCCGACGACCTCACCCGCAGTGC	A	[37]
			G759	GAGGCCGCCCTGGATCTTCGAGACGAC		
	2nd	511	B-F	GAACGAACGAGATCGAGGTCCG	B	
			B-R	CTCGACGAGCTTCGTGTT		
*gdh*	1st	n.g.	GDHeF	TCAACGTYAAYCGYGGYTTCCGT	A	[37]
			GDHeR	GTTRTCCTTGCACATCTCC		
	2nd	432	GDHiF	CAGTACAACTCYGCTCTCGG	C	
			GDHiR	GTTRTCCTTGCACATCTCC		
*18S* rRNA	1st	292	RH11	ATCCGGTCGATCCTGCC	A	[37]
			RH4	AGTCGAACCCTGATTCTCCGCCAGG		
	2nd	130	GiarF	GACGCTCTCCCCAAGGAC	B	
			GiarR	TGCGTCACGCTGCTCG		
*tpi*	1st	605	ALA3542	AAATIATGCCTGCTCGTCG	A	[38]
			ALA3542	CAAACCTTITCCGCAAACC		
	2nd	530	ALA3544	CCCTTCATCGGIGGTAACTT		
			ALA3545	GTGGCCACCACICCCGTGCC		

*Notes*: PCR conditions: A (1 cycle: 95 °C for 5 min; 40 cycles: 95 °C for 45 s, 50 °C for 30 s, 72 °C for 60 s; 1 cycle: 72 °C for 7 min); B (1 cycle: 95 °C for 5 min; 35 cycles: 95 °C for 45 s, 55 °C for 30 s, 72 °C for 45 s; 1 cycle: 72 °C for 7 min); C (1 cycle: 95 °C for 5 min; 40 cycles: 95 °C for 45 s, 60 °C for 30 s, 72 °C for 45 s; 1 cycle: 72 °C for 7 min)

Agarose gel (1.5%) electrophoresis, stained with SYBR Safe DNA gel stain (Invitrogen, California, US), was performed for the visualization of PCR products. Positive and negative controls were included in each PCR reaction set and DNA extraction.

### DNA sequencing

The PCR products were purified by using a QIAquick PCR purification kit (Qiagen, Hilden, Germany) and sequenced (Macrogen Europe, Amsterdam, Netherlands). Nucleotide sequence data from this study were submitted to the GenBank database under the following accession numbers: MN457734–MN457735; MN457739–MN457741; MT060490–MT060492; MT078609; MT001293; and MT060487–MT060489. Nucleotide sequences were aligned with all homologous sequences (> 99% similarity) available in GenBank using the Basic Local Alignment Search Tool (BLAST).

### Statistical analysis

Data analysis was performed using the EpiInfo 2000 software (CDC, Atlanta, GA, USA) and VassarStats (Website for Statistical Computation; http://vassarstats.net).

### Phylogenetic analysis

Because there are very limited intra-assemblage variations in ITS sequences, phylogenetic analysis of sequences at this locus was not performed in the present study. The phylogenetic trees were obtained using sequences of the *tpi*, *gdh* and *bg* genes available in GenBank of *G. duodenalis* species isolated from feces (host *Homo sapiens*). Phylogenetic analysis was performed with MEGA X software [36]. The evolutionary history was inferred using the Neighbor-Joining method. The bootstrap consensus tree inferred from 1000 replicates is taken to represent the evolutionary history of the taxa analyzed. Branches corresponding to partitions reproduced in less than 50% bootstrap replicates are collapsed. The percentage of replicate trees in which the associated taxa clustered together in the bootstrap test (1000 replicates) are shown above the branches. The evolutionary distances were computed using the Kimura 2-parameter model and are in the units of the number of base substitutions per site. The sequences from Romanian isolates were aligned using reference sequences of *G. duodenalis* (as *G. lamblia*) from GenBank.

## Results

Among the total number of 7805 patients included in the study, 33 (0.42%; 95% CI: 0.3–0.59%) tested positive for *G. duodenalis* by optical microscopy. PCR analysis and sequencing confirmed 30 (0.38%; 95% CI: 0.27–0.55%) *Giardia*-positive samples. Amplification of 3 fecal samples were weak, thus these samples were unsuccessfully sequenced. Representative sequences were submitted to the GenBank database. GenBank accession numbers are presented in Table 2.

**Table 2:** GenBank accession numbers for *tpi*, *gdh* and *bg* genes used for sequence analysis and in the phylogenetic tree construction

Gene	Assemblage	Subtype	GenBank ID
*bg*	A	A2	AY072723, FN386482, MN457734
		A3	MN457735
		A2/A3	MN457741
		Not specified	JQ978667, MN457740
	B	na	AY072726, AY072728, EU014384, EU216429, KP026314, KP026313, HM165226, LC437422, MN457739
	C		LC437440
	D		LC437467
	E		KC960635
	F		LC341557
	Outgroup		AY258618
*gdh*	A	A1	AB434776, EF507606
		A2	AB195222, AY178735, AY178736, AY178737, AY826194, EF507674, L40510, M84604, MT078609
		A3	MT060492
		Not specified	EU637582, KM190756
	B	na	AB434535, AF069059, AY178738, AY826191, DQ090539, DQ090540, EF685684, EU594665, KP899844, L40508, MT060490, MT060491
	C		U60984
	D		U60986
	E		KC960651
	F		AF069057
	G		AF069058
	Outgroup		AF069060
*tpi*	A	A1	GU564274
		A2	AB516350, L02120, KC313923, KR105400, KR902356, KY271716, KY271722, MT060487, U57897
		A3	MT060488
		Not specified	DQ650648, KJ941325, KM190773, KT369760, KU378623, MT001293
	B	na	AF069560, AF069561, AY368163, AY368167, GU564279, HM140722, KC632557, KF679740, KF843920, KT357495, KT948104, KY271715, KY271717, MT060489
	C		AY228641
	D		DQ246216
	E		KF891311
	G		EU781013
	Outgroup		AF069564

*Abbreviation*: na, not applicable

### Molecular genotyping

Of the 30 samples submitted for genotyping 100% were successfully genotyped at least at three or four loci. Twenty-three samples (76.67%; 95% CI: 57.72–90.07%) were successfully genotyped at each locus, while 7 samples (23.33%; 95% CI: 9.93–42.28%) were genotyped at three loci. The *bg* and *tpi* genes had the highest typing success rate (100%; 95% CI: 83.43–100%), followed by *gdh* gene (93.33%; 95% CI: 77.93–99.18%), and lastly, the ITS sequence presenting an 83.33% (95% CI: 65.28–94.36%) success rate (Table 3). Data for reference sequences used in the sequence alignment and analysis are provided in Table 2.

**Table 3:** Occurrence of sequence variants at each locus in *G. duodenalis* isolates

Locus	A	A2	A3	A2/A3	B	Total
ITS	1	14	4	3	3	25
*gdh*	1	22	1	1	3	28
*bg*	1	22	2	2	3	30
*tpi*	1	25	1	0	3	30

#### Molecular typing of the ITS region

Amplification of the ITS-positive samples was obtained in 25 samples. Sequence analysis revealed assemblages A (73.33%) and B (10%); sub-assemblage AII was recorded in 21 (70%) out of 30 PCR-positive samples, whereas subtypes A2 (46.67%) and A3 (13.33%) were identified. Sequence analysis of 3 (10%) of the samples showed equal degree similarity with sequences with subtype A2 and A3, thus these sequences were identified as A2/A3 (Table 4). The BLAST analysis at each locus for one of the samples showed a low degree of identity with reference sequences and was identified as assemblage A without subtype identification.

**Table 4:** Genotyping data of each sample at different loci

Sample	ITS	*gdh*	*bg*	*tpi*
1	A2	A2	A2	A2
2	A2	A2	A2	A2
3	A2	A2	A2	A2
4	A2/A3	A2	A2/A3	A2
5	A2	A2	A2	A2
6	A2	A2	A2	A2
7	A2/A3	–	A2	A2
8	B	B	B	B
9	B	B	B	B
10	A3	–	A2	A2
11	A2	A2	A2	A2
12	A3	A2	A2	A2
13	–	A2	A2	A2
14	A2	A2	A2	A2
15	A2	A2	A2	A2
16	A2	A2	A2	A2
17	A2	A2	A3	A2
18	A2	A2	A2	A2
19	A2	A2	A2	A2
20	B	B	B	B
21	A3	A2	A2	A2
22	A2/A3	A2/A3	A2/A3	A2
23	A3	A3	A3	A3
24	A	A	A	A
25	–	A2	A2	A2
26	–	A2	A2	A2
27	A2	A2	A2	A2
28	A2	A2	A2	A2
29	–	A2	A2	A2
30	–	A2	A2	A2

#### Molecular typing of the gdh gene

Amplification of the *gdh*-positive samples was obtained in 28 (93.33%) out of 30 samples (Table 3). Sequence analysis revealed assemblages A (25/30, 83.33%) and B (3/30, 10%). Twenty-four (80%) isolates were identified as sub-assemblage AII, from which 22 (73.33%) isolates showed complete sequence identity with subtype A2, while 1 (33.3%) with subtype A3. Equal degrees of similarity with subtypes A2 and A3 were identified in one of the samples (3.33%) (Table 4).

#### Molecular typing of the bg gene

Amplification of the *bg* gene was successfully obtained in all of the samples. Sequence analysis revealed assemblages A (27/30, 90%) and B (3/30, 10%). In total, 26 (86.67%) isolates showed complete sequence identity with sub-assemblage AII, while subtype A2 (22/30, 73.33%) and subtype A3 (2/30, 6.67%) were identified (Table 3). Equal degree identity of infections with subtype A2 and A3 were found in 2 (6.67%) of the samples (Table 4).

#### Molecular typing of the tpi gene

Amplification of the *tpi* gene was successfully obtained in all of the samples. Assemblages A (27/30, 90%) and B (3/30, 10%) were identified. Sequence analysis revealed sub-assemblage AII in 26 (86.66%) samples. Twenty-five (83.33%) isolates showed complete sequence identity with subtype A2, while 1 (3.33%) isolate with subtype A3. The sub-assemblage of 1 of the sample could not be determined, since its sequence did not show similarity to assemblage A reference sequences (Table 4).

### Phylogenetic analysis

**Fig. 1:**
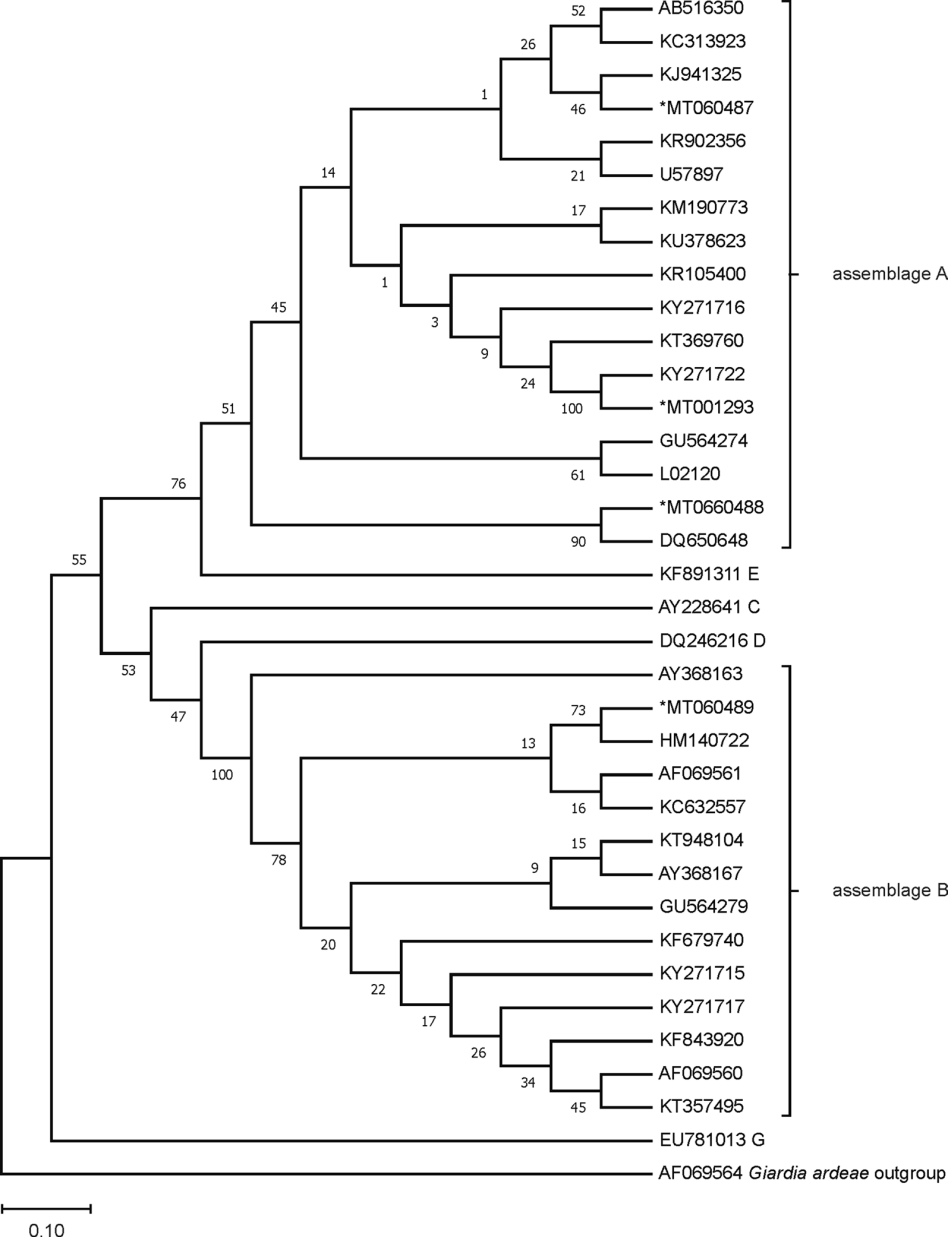
Phylogenetic tree constructed for the *tpi* gene sequences of *G. duodenalis* isolates. The evolutionary distances were computed using the Kimura 2-parameter method and are in the units of the number of base substitutions per site. Evolutionary analyses were conducted in MEGA X software and further bootstrap analysis of 1000 replicas. Sequences obtained from GenBank are indicated by their accession numbers. Values at the nodes represent bootstrap support

**Fig. 2:**
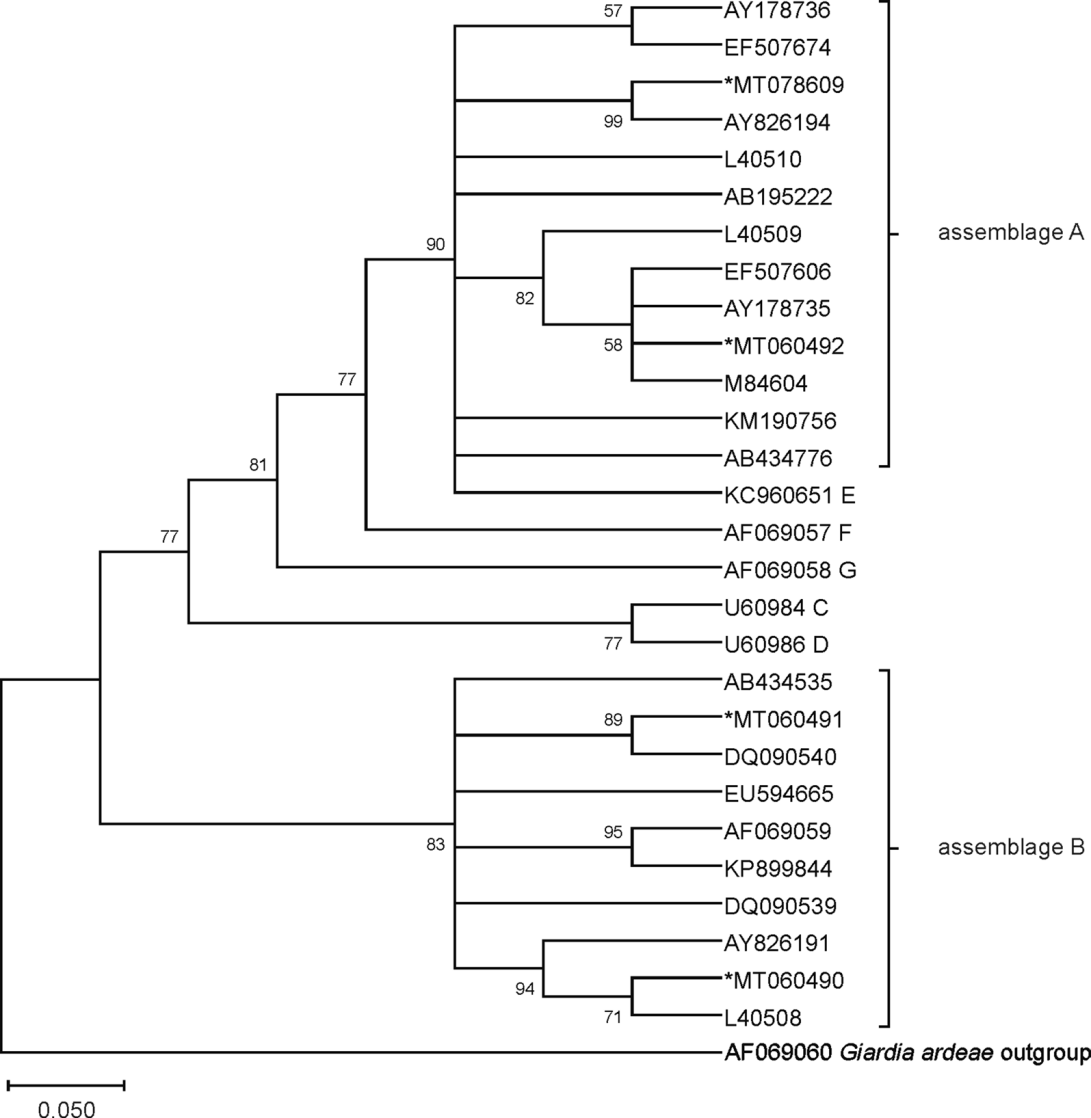
Phylogenetic tree constructed for the *gdh* gene sequences of *G. duodenalis* isolates. The evolutionary distances were computed using the Kimura 2-parameter method and are in the units of the number of base substitutions per site. Evolutionary analyses were conducted in MEGA X software and further bootstrap analysis of 1000 replicas. Sequences obtained from GenBank are indicated by their accession numbers. Values at the nodes represent bootstrap support

**Fig. 3:**
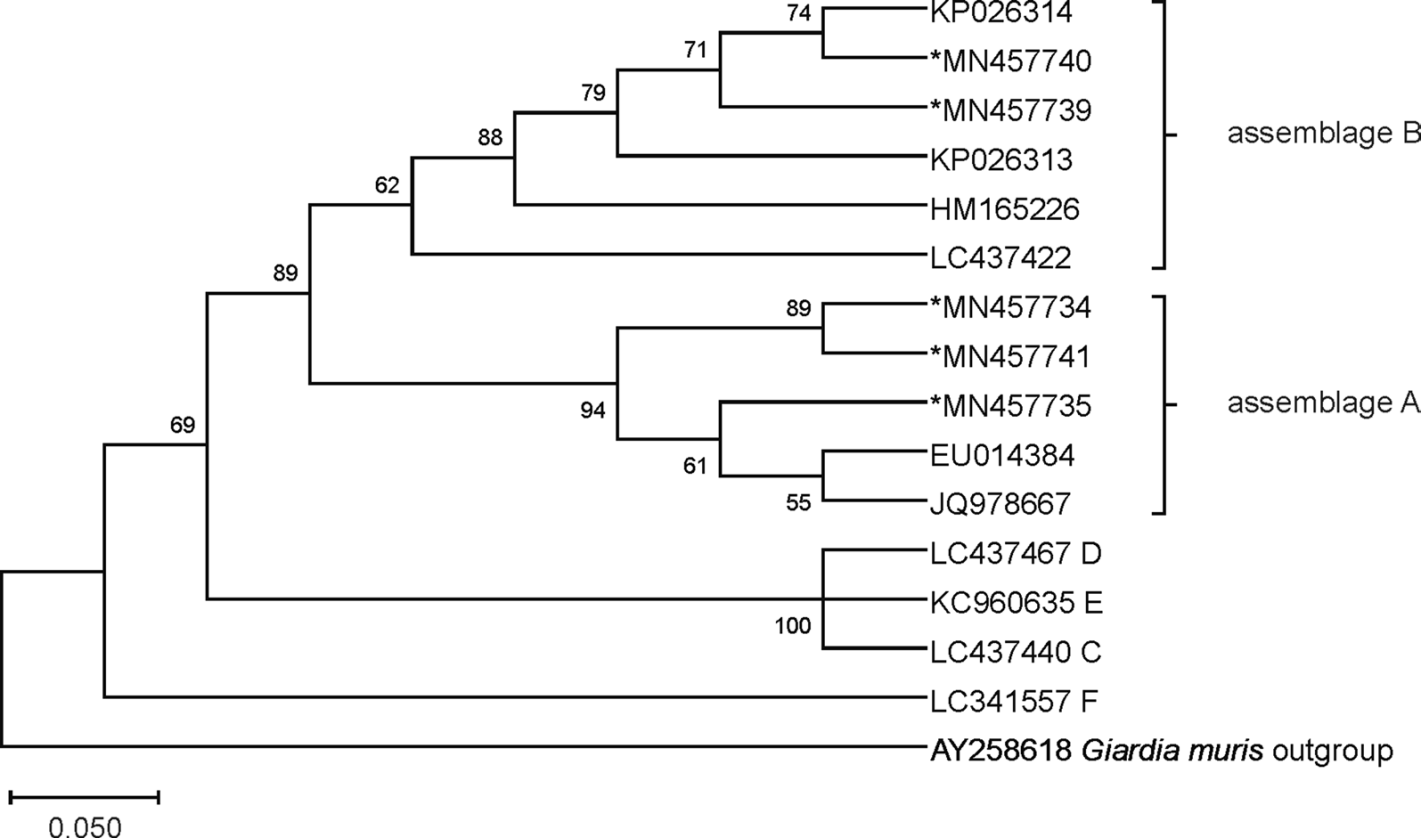
Phylogenetic tree constructed for the *bg* gene sequences of *G. duodenalis* isolates. The evolutionary distances were computed using the Kimura 2-parameter method and are in the units of the number of base substitutions per site. Evolutionary analyses were conducted in MEGA X software and further bootstrap analysis of 1000 replicas. Sequences obtained from GenBank are indicated by their accession numbers. Values at the nodes represent bootstrap support

Figures 1, 2 and 3 include the phylogenetic trees with the relative position of *G. duodenalis* isolates from Romania for *bg*, *gdh* and *tpi* genes. For all the genes, sequences were grouped in two distinct lineages, in assemblages A and B. Subtypes A2/A3 were identified in 2 of the samples at *bg* and *gdh* loci and are closely related with the sequences with A2 subtypes.

## Discussion

In Romania, studies that focused on the molecular diversity of the parasite were conducted on samples obtained from animals [26, 27, 33], while *Giardia* infections in humans were analyzed from an epidemiological point of view, focusing on prevalence and symptomology [5, 34]. Genotyping studies of human isolates are lacking in our country. To fill in these blanks, the main goal achieved by this study was to investigate the genotypes of *G. duodenalis* isolated from human fecal samples taken in Romania.

The data published by the European Center for Disease Control (ECDC) from Romania in 2017 reported 1060 confirmed cases; however, the notification rate was not calculated because the national surveillance system is sentinel and does not cover the whole population [35]. Other countries in Europe reported lower prevalence than our study, in routine check-ups of the population: 0.07% in Croatia and 0.28 % in Serbia, while Hungary and Slovenia reported slightly higher percentages at 1.2% and 0.96%, respectively [12].

Molecular characterization of *G. duodenalis* isolates is an important step for public health, necessary for the discrimination of the zoonotic assemblages (A and B) [37–39]. To understand the zoonotic linkage, a multilocus genotyping approach is suggested. In the present study, MLST was performed, targeting four genetic loci, the *gdh*, *tpi*, *bg* genes and the ITS region. Whereas the sensitivity of PCR targeting the ITS region is known, its intra-assemblage and sub-genotype variation is limited. The *bg* and *gdh* genes are frequently used discriminatory markers, thus may show more intra-assemblage variation [15]. The amplification success rate in the present study was 100% at the *bg* and *tpi* loci, and lower at the *gdh* locus and ITS region. Despite repeating molecular analysis, sequencing failure occurred at the ITS region in five of the samples, and at *gdh* locus in two samples, respectively.

Regarding the molecular diversity of *G. duodenalis*, the present study found that the majority of human infections were caused by assemblage A, with most isolates successfully characterized at the subtype level. More than three-quarters of assemblage A isolates pertained to the A2 subtype, with most of the sequences matching previously described isolates. Our analysis found only a small number of A3 (mainly at the ITS region) and no A1 subtype, thus reinforcing the status of A2 as the main A subtype found in humans, as previously demonstrated by other MLST studies [40, 41]. The high variation of assemblage B at the *bg*, *tpi* and *gdh* loci yield to an inconsistent typing result of assemblage B [42]. Three samples were identified as assemblage B at each locus. However, sequence analysis showed complete sequence identity with the B3 subtype reference sequence (GenBank: AF069561) at the *tpi* locus [40], while at the other three loci, the subtype could not be unequivocally determined.

The phylogenetic tree provides an overview of the phylogenetic situation of the *G. duodenalis* isolates from human feces collected in Romania. Sequencing of the isolates from this study at each locus, and phylogenetic analysis of these sequences with a large set of available sequences in the GenBank database, showed highly genetic homogeneity (99–100%) with the published sequences. Because of the high sequence heterogeneity of the *tpi* gene, sequences of this gene provided greater sensitivity in subtype of assemblage A differentiation. However, analysis of the BLAST search of *tpi*-sequences revealed 7 isolates with subtype A2 of those sequences that were identified as A2/A3 (3 isolates) and A3 (4 isolates) subtypes on *gdh* (1 isolate with A2/A3 subtype), *bg* (2 isolates with A2/A3 and 1 with A3 subtype) genes and ITS sequence (3 isolates with A2/A3 and 2 with A3 subtype) (Table 3). The detected difference in the results of one sample at multiple loci (where two different assemblages were identified with the same similarity rate) may be due to the use of primers that may lead to imperfect discrimination between two assemblages within the same isolate (to detect mixed infections) or unsuccessful sequencing (low sequence similarity). However, discrepancies in assemblage type were observed by sequencing of the ITS region and the *bg* gene that led to an inability to group all the isolates into A2 or A3 subtypes. Even an additional PCR and chromatogram analysis of isolates with the A2/A3 subtype could not discriminate between these subtypes.

In Romania, the assemblages B, D and E were found in farmed long-tailed chinchillas [26] and C, D and E in domestic and wild animals [27, 33]. In the present study, we identified two assemblages, A and B, however the occurrence of sequence variants at each locus revealed different subtypes of sub-assemblage AII (A2 and A3). Sub-assemblage AII is known to be more frequent in human isolates [19, 43], while the identification of sub-assemblage AIII was reported to infect mainly hoofed wild animals [15, 42]. The difference between the types of assemblages identified in samples from domestic and wild animals in our area (B, C, D and E) and those from human samples (A and B) may suggest an anthroponotic transmission rather than a zoonotic one, especially in regards to assemblage A, which was overwhelmingly more common than B (27 *vs* three). Although more data is required for a clear image of possible infection routes, the lack of sub-assemblage AI (mainly found in livestock and pets) in our results also supports this hypothesis [44]. In contrast with our results, several studies worldwide have reported a higher frequency of human infections with assemblage B [9]. In four studies carried out in Spain, human infections with assemblage B were more frequent than those with assemblage A [21, 45–47]; assemblage B was also found to be the most prevalent (74.4%) in Belgium [41]. However, in a study conducted in the UK the distribution of the assemblages A and B were different in relation to age group; equal distribution in children, assemblage B more common in young adults and assemblage A more common in adults over 50 [40]. Previous studies conducted in Rio de Janeiro, Brazil, found only assemblage A isolates, with the first assemblage B reported in 2016 [48]. All the samples from the present study originated from adults, and were predominantly identified as assemblage A. Similar results were reported in other studies conducted in UK, Rio de Janeiro (Brazil), Canada and South Korea [40, 48].

The association between different assemblages and clinical outcome is still not clear, even though a large number of studies have been published on this subject [9, 18]. Regarding the clinical outcome, in our study we did not have the possibility to thoroughly analyze this aspect. However, because samples were acquired from presumably asymptomatic patients (they were collected during regular check-ups of employees) and 90% (27/30) of them were assemblage A, we can hypothesize that in our study, similar to interpretation from other studies, patients infected with assemblage A are more likely to be asymptomatic [19, 49–51].

Differentiation of genotypes circulating in a geographic area is a useful tool for the understanding of giardiasis epidemiology within that area, an important basis for effective prevention methods. Further studies on the molecular diversity of *G. duodenalis* isolated from symptomatic patients in Romania are required in order to comprehensively understand the epidemiology of giardiasis in our country.

## Conclusions

The prevalence of asymptomatic infection with *G. duodenalis* in adults from our area was 0.42%. This study has produced the first molecular characterization of *G. duodenalis* isolated from human fecal samples in Romania. The majority of infections were caused by assemblage A, subtype A2. All four loci showed a high typing success rate, with the *tpi* gene being the most profitable marker for genotyping and sub-assemblage discrimination.

## Data Availability

The data supporting the conclusions of this article are included in the article. Representative sequences were submitted to the GenBank database under the accession numbers MN457734-MN457735, MN457739-MN457741, MT060490-MT060492, MT078609, MT001293 and MT060487-MT060489.

## Abbreviations

gdh: glutamate dehydrogenase
*bg*: β-giardin
ITS: internal transcribed spacer
*n*: number of identified samples

## References

[1] Havelaar AH, Kirk MD, Torgerson PR, Gibb HJ, Hald T, Lake RJ (2015). World Health Organization global estimates and regional comparisons of the burden of foodborne disease in 2010. PLoS Med..

[2] FAO/WHO. Multicriteria-based ranking for risk management of food-borne parasites. Microbiolological risk assessment series no. 23. Rome: Food and Agriculture Organization of the United Nations/World Health Organization; 2014.

[3] Kotloff KL, Nataro JP, Blackwelder WC, Nasrin D, Farag TH, Panchalingam S (2013). Burden and aetiology of diarrheal disease in infants and young children in developing countries (the Global Enteric Multicenter Study, GEMS): a prospective, case-control study. Lancet.

[4] Lanes S, Lloyd D (2002). Current trends in research into the waterborne parasite *Giardia*. Crit Rev Microbiol..

[5] Neghina R, Dumitrascu V, Neghina AM, Vlad DC, Petrica L, Vermesan D (2013). Epidemiology of ascariasis, enterobiasis and giardiasis in a Romanian western county (Timis), 1993–2006. Acta Trop..

[6] Olson RCA (2004). Update on *Cryptosporidium* and *Giardia* infections in cattle. Trends in Parasitol..

[7] Meyer EA, Radulescu S (1979). *Giardia* and giardiasis. Adv Parasitol..

[8] Ryan U, Hijjawi N, Feng Y, Xiao L (2019). *Giardia*: an under-reported foodborne parasite. Int J Parasitol..

[9] Einarsson E, Ma’ayeh S, Svärd SG. An up-date on *Giardia* and giardiasis. Curr Opin Microbiol. 2016;34:47–52. 10.1016/j.mib.2016.07.019 27501461

[10] Bartelt LA, Sartor RB. Advances in understanding *Giardia*: determinants and mechanisms of chronic sequelae. F1000Prime Rep. 2015;7:62. 10.12703/P7-62 PMC4447054 26097735

[11] Penrose AS, Wells EV, Aiello AE (2007). Infectious causation of chronic disease: examining the relationship between *Giardia lamblia* infection and irritable bowel syndrome. World J Gastroenterol..

[12] Plutzer J, Lassen B, Jokelainen P, Djurković-Djaković O, Kucsera I, Dorbek-Kolin E (2018). Review of *Cryptosporidium* and *Giardia* in the eastern part of Europe, 2016. Euro Surveill..

[13] Thompson RC (2000). Giardiasis as a re-emerging infectious disease and its zoonotic potential. Int J Parasitol..

[14] Hellard ME, Sinclair MI, Hogg GG, Fairley CK (2000). Prevalence of enteric pathogens among community based asymptomatic individuals. J Gastroenterol Hepatol..

[15] Lebbad M, Mattsson JG, Christensson B, Ljungström B, Backhans A, Andersson JO (2010). From mouse to moose: multilocus genotyping of *Giardia* isolates from various animal species. Vet Parasitol..

[16] Hillman A, Ash A, Elliot A, Lymbery A, Perez C, Thompson RCA (2016). Confirmation of a unique species of *Giardia*, parasitic in the quenda (*Isoodon obesulus*). Int J Parasitol Parasites Wildl..

[17] Lyu Z, Shao J, Xue M, Ye Q, Chen B, Qin Y (2018). A new species of *Giardia* Künstler, 1882 (Sarcomastigophora: Hexamitidae) in hamsters. Parasit Vectors..

[18] Ryan U, Cacciò SM (2013). Zoonotic potential of *Giardia*. Int J Parasitol..

[19] Feng Y, Xiao L (2011). Zoonotic potential and molecular epidemiology of *Giardia* species and giardiasis. Clin Microbiol Rev..

[20] Ankarklev J, Franzén O, Peirasmaki D (2015). Comparative genomic analyses of freshly isolated *Giardia* intestinalis assemblage A isolates. BMC Genomics..

[21] Wang Y, Gonzalez-Moreno O, Roellig DM, Oliver L, Huguet J, Guo Y (2019). Epidemiological distribution of genotypes of *Giardia duodenalis* in humans in Spain. Parasit Vectors..

[22] Franzén O, Jerlström-Hultqvist J, Castro E, Sherwood E, Ankarklev J, Reiner DS, Palm D (2009). Draft genome sequencing of *Giardia intestinalis* assemblage B isolate GS: is human giardiasis caused by two different species?. PLoS Pathog..

[23] Caccio SM, Thompson RC, McLauchlin J, Smith HV (2005). Unraveling *Cryptosporidium* and *Giardia* epidemiology. Trends Parasitol..

[24] Tak V, Mirdha BR, Yadav P, Makharia GK, Bhatnagar S (2014). Molecular characterization of *Giardia intestinalis* assemblages from human isolates at a tertiary care center of India. Indian J Med Microbiol..

[25] Steriu D. Parasitic infections (in Roumanian: infectii parazitare). Bucharest: Ilex Ed; 2003. p. 30–2.

[26] Gherman CM, Kalmár Z, Györke A, Mircean V (2018). Occurrence of *Giardia duodenalis* assemblages in farmed long-tailed chinchillas *Chinchilla lanigera* (Rodentia) from Romania. Parasit Vectors..

[27] Adriana G, Zsuzsa K, Mirabela Oana D, Mircea GC, Viorica M (2016). *Giardia duodenalis* genotypes in domestic and wild animals from Romania identified by PCR-RFLP targeting the *gdh* gene. Vet Parasitol..

[28] Imre K, Sala C, Morar A, Ilie MS, Plutzer J, Imre M, et al. *Giardia duodenalis* and *Cryptosporidium* spp. as contaminant protozoa of the main rivers of western Romania: genetic characterization and public health potential of the isolates. Environ Sci Pollut Res Int. 2017;24:18672–9. 10.1007/s11356-017-9543-y 28653194

[29] Hiatt RA, Markell EK, Ng E (1995). How many stool examinations are necessary to detect pathogenic intestinal protozoa?. Am J Trop Med Hyg..

[30] Garcia LS, Arrowood M, Kokoskin E, Paltridge GP, Pillai DR, Procop GW, Ryan N, Shimizu RY, Visvesvara G (2017). Laboratory diagnosis of parasites from the gastrointestinal tract. Clin Microbiol Rev..

[31] Hooshyar H, Rostamkhani P, Arbabi M, Delavari M (2019). *Giardia lamblia* infection: review of current diagnostic strategies. Gastroenterol Hepatol Bed Bench..

[32] Papaiakovou M, Pilotte N, Baumer B, Grant J, Asbjornsdottir K, Schaer F (2018). A comparative analysis of preservation techniques for the optimal molecular detection of hookworm DNA in a human fecal specimen. PLoS Negl Trop Dis..

[33] Onac D, Oltean M, Mircean V, Jarca A, Cozma V (2015). Occurrence of *Giardia duodenalis* zoonotic assemblages in red foxes from Romania. Sci Parasitol..

[34] Costache C, Colosi I, Anca L. Human giardiasis report in Romania: the principle of snowball. Proceedings of the XIth European Multicolloquium of Parasitology, 25–29 July 2012, Cluj-Napoca, Romania; 2012.

[35] ECDC. Giardiasis (lambliasis). In: ECDC. Annual epidemiological report for 2017. Stockholm: European Centre for Disease Prevention and Control; 2019. p. 2.

[36] Kumar S, Stecher G, Li M, Knyaz C, Tamura K (2018). MEGA X: Molecular evolutionary genetics analysis across computing platforms. Mol Biol Evol..

[37] Gillhuber J, Pallant L, Ash A, Thompson RC, Pfister K, Scheuerle MC (2013). Molecular identification of zoonotic and livestock-specific *Giardia*-species in faecal samples of calves in southern Germany. Parasit Vectors..

[38] Cacció SM, Beck R, Lalle M, Marinculic A, Pozio E (2008). Multilocus genotyping of *Giardia duodenalis* reveals striking differences between assemblages A and B. Int J Parasitol..

[39] Monis PT, Andrews RH, Mayrhofer G, Ey PL (2003). Genetic diversity within the morphological species *Giardia intestinalis* and its relationship to host origin. Infect Gen Evol..

[40] Minetti C, Lamden K, Durband C, Cheesbrough J, Fox A, Wastling JM (2015). Determination of *Giardia duodenalis* assemblages and multi-locus genotypes in patients with sporadic giardiasis from England. Parasit Vectors..

[41] Geurden T, Levecke B, Cacció SM, Visser A, De Groote G, Casaert S (2009). Multilocus genotyping of *Cryptosporidium* and *Giardia* in non-outbreak related cases of diarrhoea in human patients in Belgium. Parasitology..

[42] Wielinga CM, Thompson RC (2007). Comparative evaluation of *Giardia duodenalis* sequence data. Parasitology..

[43] Puebla LJ, Fidel AN, Yenisey AF, Jorge F, Lázara RR, Iraís AM (2014). Correlation of *Giardia duodenalis* assemblages with clinical and epidemiological data in Cuban children. Infect Gen Evol..

[44] Sprong H, Caccio SM, van der Giessen JW (2009). Identification of zoonotic genotypes of *Giardia duodenalis*. PLoS Negl Trop Dis..

[45] de Lucio A, Martinez-Ruiz R, Merino FJ, Bailo B, Aguilera M, Fuentes I (2015). Molecular genotyping of *Giardia duodenalis* isolates from symptomatic individuals attending two major public hospitals in Madrid. Spain. PLoS ONE..

[46] Azcona-Gutierrez JM, de Lucio A, Hernandez-de-Mingo M, Garcia-Garcia C, Soria-Blanco LM, Morales L, et al. Molecular diversity and frequency of the diarrheagenic enteric protozoan *Giardia duodenalis* and *Cryptosporidium* spp. in a hospital setting in northern Spain. PLoS ONE. 2017;12:e0178575. 10.1371/journal.pone.0178575 PMC5472271 28617836

[47] Sahagun J, Clavel A, Goni P, Seral C, Llorente MT, Castillo FJ (2008). Correlation between the presence of symptoms and the *Giardia duodenalis* genotype. Eur J Clin Microbiol Infect Dis..

[48] Faria CP, Zanini GM, Dias GS, da Silva S, Sousa MdC. Molecular characterization of *Giardia lamblia*: first report of assemblage B in human isolates from Rio de Janeiro (Brazil). PLoS ONE. 2016;11:e0160762. 10.1371/journal.pone.0160762 PMC4982690 27517469

[49] Gelanew T, Lalle M, Hailu A, Pozio E, Caccio SM (2007). Molecular characterization of human isolates of *Giardia duodenalis* from Ethiopia. Acta Trop..

[50] Homan WL, Mank TG (2001). Human giardiasis: genotype linked differences in clinical symptomatology. Int J Parasitol..

[51] Molina N, Minvielle M, Grenovero S, Salomon C, Basualdo J (2011). High prevalences of infection with *Giardia intestinalis* genotype B among children in urban and rural areas of Argentina. Ann Trop Med Parasitol..

