# Correction to: First multilocus sequence typing (MLST) of Giardia duodenalis isolates from humans in Romania

**DOI:** 10.1186/s13071-021-04822-2

**Published:** 2021-06-15

**Authors:** Carmen Costache, Zsuzsa Kalmár, Horațiu Alexandru Colosi, Alina Mihaela Baciu, Răzvan Vlad Opriş, Adriana Györke, Ioana Alina Colosi

**Affiliations:** 1 Department of Molecular Sciences, Discipline of Microbiology, Iuliu Hațieganu University of Medicine and Pharmacy, 6 Louis Pasteur Street, 400349 Cluj-Napoca, Romania; 2 Department of Parasitology and Parasitic Diseases, Faculty of Veterinary Medicine, University of Agricultural Sciences and Veterinary Medicine Cluj-Napoca, 3–5 Calea Mănăştur, 400372 Cluj-Napoca, Romania; 3 Department of Medical Education, Discipline of Medical Informatics and Biostatistics, Iuliu Hațieganu University of Medicine and Pharmacy, 6 Louis Pasteur Street, 400349 Cluj-Napoca, Romania

## Correction to: Parasites Vectors 13, 387 (2020) https://doi.org/10.1186/s13071-020-04248-2

Following publication of the original article [1], it was brought to our attention that incorrect primers information had been provided in Table 1.

**Table 1:** Primers and PCR conditions

Gene	PCR reaction	Product length (bp)	Primer name	Primer (5'-3')	PCR conditions	References
*bg*	1st	753	G7	AAGCCCGACGACCTCACCCGCAGTGC	A	[37]
			G759	GAGGCCGCCCTGGATCTTCGAGACGAC		
	2nd	511	B-F	GAACGAACGAGATCGAGGTCCG	B	
			B-R	CTCGACGAGCTTCGTGTT		
*gdh*	1st	n.g	GDHeF	TCAACGTYAAYCGYGGYTTCCGT	A	[37]
			GDHeR	GTTRTCCTTGCACATCTCC		
	2nd	432	GDHiF	CAGTACAACTCYGCTCTCGG	C	
			GDHiR	GTTRTCCTTGCACATCTCC		
ITS1	1st	347	FW1	TGGAGGAAGGAGAAGTCGTAAC	D	[16]
			RV1	GGGCGTACTGATATGCTTAAGT		
	2nd	315	FW2	AAGGTATCCGTAGGTGAACCTG		
			RV2	ATATGCTTAAGTTCCGCCCGTC		
*tpi*	1st	605	ALA3542	AAATIATGCCTGCTCGTCG	A	[38]
			ALA3542	CAAACCTTITCCGCAAACC		
	2nd	530	ALA3544	CCCTTCATCGGIGGTAACTT		
			ALA3545	GTGGCCACCACICCCGTGCC		

PCR conditions: A (1 cycle: 95 °C for 5 min; 40 cycles: 95 °C for 45 s, 50 °C for 30 s, 72 °C for 60 s; 1 cycle: 72 °C for 7 min); B (1 cycle: 95 °C for 5 min; 35 cycles: 95 °C for 45 s, 55 °C for 30 s, 72 °C for 45 s; 1 cycle: 72 °C for 7 min); C (1 cycle: 95 °C for 5 min; 40 cycles: 95 °C for 45 s, 60 °C for 30 s, 72 °C for 45 s; 1 cycle: 72 °C for 7 min); D (1 cycle: 95 °C for 5 min; 35 cycles: 95 °C for 30 s, 59 °C for 30 s, 72 °C for 30 s; 1 cycle: 72 °C for 7 min)

The table has since been corrected and the updated table is provided in this correction for reference.

The authors apologize for any inconvenience caused.
